# An Array Switching Strategy for Direction of Arrival Estimation with Coprime Linear Array in the Presence of Mutual Coupling

**DOI:** 10.3390/s20061629

**Published:** 2020-03-14

**Authors:** Jinqing Shen, Yi He, Jianfeng Li

**Affiliations:** 1 College of Electronic and Information Engineering, Nanjing University of Aeronautics and Astronautics, Nanjing 211106, China; 2 Key Laboratory of Dynamic Cognitive System of Electromagnetic Spectrum Space, Ministry of Industry and Information Technology, Nanjing 211106, China

**Keywords:** direction of arrival, coprime array, mutual coupling, array switching

## Abstract

While the coprime array still suffers from performance degradation due to the mutual coupling dominated by the interleaved subarrays, we propose an array switching strategy for coprime linear array (CLA) by utilizing the large inter-element spacings of the subarrays to mitigate the mutual coupling. Specifically, we first collect the signals by separately activating the two subarrays, where the severe mutual coupling effect is significantly reduced. As a result, well-performed initial direction of arrival (DOA) estimates can be achieved. Subsequently, we establish a quadratic optimization problem by reconstructing the contaminated steering vector of the total CLA elaborately to calculate the mutual coupling coefficients with the initial DOA estimates. Finally, we can obtain refined DOA estimates by an iteration procedure based on the estimated mutual coupling matrix. In addition, numerical simulations are provided to demonstrate the merits of the proposed scheme.

## 1. Introduction

As a fundamental part of array signal processing, direction of arrival (DOA) estimation plays a critical role in various fields, e.g., radar, acoustics, cognitive radio and wireless communication [1,2,3,4,5,6,7]. Numerous high-resolution DOA estimation algorithms are developed for uniform linear arrays (ULAs), such as MUSIC [8], ESPRIT [9] and the variants [10,11]. However, the compact array structure will lead to limited array aperture and serious mutual coupling effect.

In recent years, coprime array [12,13,14,15,16] has received considerable attention due to the promising merits, such as the enhanced resolution, the increased degrees of freedom (DOFs) and the reduced mutual coupling. Specifically, the DOA estimation methods proposed for coprime linear array (CLA) can be divided into two categories in general. The first type is to decompose the CLA into two uniform subarrays and then eliminate the phase ambiguity problem based on the arrangements with coprime property [17,18,19]. The other one is to construct an equivalent received signal collected from an augmented virtual array to avoid spatial aliasing and enhance the DOF [20,21,22,23]. However, the aforementioned researches critically depend on the array manifold that is not distorted by impairments such as mutual coupling [24]. Since the two subarrays in the prototype CLA are interleaved, the mutual coupling cannot be neglected, which can significantly degrade the performance. To tackle this problem, an unfolded CLA was designed in [25], where the two subarrays are configured along the positive and negative axis respectively and thereby the minimum adjacent distance is enlarged. The coprime array with displaced subarrays (CADiS) in [26] introduces a displacement between the two subarrays to enlarge the smallest inter-element spacing, which can prominently alleviate the mutual coupling. Particularly, an extended coprime array structure in [27] relocates a proper number of sensors in the CADiS, where the mutual coupling is further reduced. In [28], the thinned coprime array exploits the redundancy in the difference co-array to achieve increased DOFs, where the mutual coupling is decreased simultaneously, owing to the reduced number of sensor pairs with small separations. However, since at least one sensor pair is needed to generate the virtual sensors, the sensor pairs with small separations still lead to severe mutual coupling effect. Although significant efforts have been put into mutual coupling reduction techniques for CLA, the investigations about mutual coupling calibration for CLA are neglected.

As alternative approaches to deal with mutual coupling, a series of mutual coupling calibration algorithms are proposed [29,30,31,32,33,34,35,36,37], and a few of them are applicable to nonuniform linear arrays. Specifically, the active calibration [35] utilizes auxiliary sources with exactly known locations to perform calibration, whereas it is time-consuming and impractical in real systems. Moreover, as the mutual coupling effect varies with the environment, repeated off-line calibrations are not conducive to the engineering applications. An iteration-based calibration method was proposed in [36] for nonuniform linear arrays, but the solution may fall into local optimum and the convergence cannot be guaranteed theoretically. Besides, two mutual coupling compensation methods for nonuniform linear arrays were proposed in [37]. The first one is an iteration method which is based on the precisely known mutual impedance matrix obtained by electromagnetic simulation while the second method is proposed by employing a global optimization algorithm, but suffering from heavy computational burden and the loss of the structural characteristics of CLA.

In this paper, the issue of DOA estimation for the prototype CLA with unknown mutual coupling is investigated and an array switching strategy is designed based on the structural features of CLA. Specifically, the two subarrays are separately activated as received arrays first, where the mutual coupling effect can be mitigated to a great extent since the coupling between subarrays is avoided. Then we exploit the uncalibrated polynomial rooting to the two subarrays respectively and obtain the initial DOA estimates by exploiting the coprime property, which is less susceptible to mutual coupling benefiting from sparse arrangement of CLA. Subsequently, the total CLA is activated to collect the signals and the contaminated steering vector is remodeled for decoupling, which is carefully extended from the reconstruction in ULA utilizing two selection matrices. Furthermore, the closed-form solutions to the mutual coupling coefficients (MCCs) are provided by employing a quadratic optimization problem. Finally, re-estimation with few iterations is performed to refine the parameter estimates. Numerical simulations corroborate that the proposed array switching-based scheme for CLA can effectively deal with the mutual coupling and achieve improved estimation accuracy.

We summarize the main contributions of our work as follows.

(1) The unknown mutual coupling in CLA potentially degrades the estimation performance, whereas the conventional calibration methods for uniform arrays are difficult to apply to CLA due to its nonuniform structure. To tackle this issue, we comprehensively investigate the characteristics of mutual coupling in CLA and significantly mitigate the mutual coupling by exploiting the inherent sparse structural features of CLA.
(2) We propose an array switching strategy, which can be developed for online calibration, by separately activating the subarrays of CLA to considerably alleviate the severe mutual coupling caused by the two interleaved subarrays in CLA and calculate the well-performed DOA estimates with the signals from subarrays based on the coprime property.
(3) We reconstruct the contaminated received signal of the total CLA to directly solve the mutual coupling coefficients by utilizing the initial DOA estimates and, in turn, calculate the refined DOA estimates via an iteration procedure. In particular, the reconstruction of the steering vector of CLA for decoupling can be extended to nonuniform linear arrays of arbitrary structure.

The remainder of the paper is given as follows. We provide the preliminary for our work in Section 2, including the data model of CLA and the analysis of mutual coupling characteristics in CLA. Section 3 elaborates the proposed scheme via the array switching strategy and the performance analysis is provided in Section 4. Section 5 exhibits numerical simulations and Section 6 concludes this paper.

*Notations*: Matrices and vectors are denoted by upper-case and lower-case bold characters, respectively. ${\left(\cdot \right)}^{T}$ and ${\left(\cdot \right)}^{H}$ are transpose and conjugate transpose operators, respectively. x∈〈a,b〉 represents the set of integers which satisfies a≤x≤b. $I_{M}$ denotes an M×M identity matrix. $0_{M\times N}$ stands for an M×N matrix in which all elements are zero. diag(C) symbolizes a matrix composed of the principal diagonal elements of C. Toeplitz{c} generates a symmetric Toeplitz matrix having c as its first column and first row. ${\left\|\cdot \right\|}_{F}$ is Frobenius norm. angle(⋅) represents the phase operator. tr(·) is the trace of matrix.

## 2. Preliminaries

In this section, we first introduce the data model of CLA in the absence of mutual coupling. Subsequently, we modify the data model with mutual coupling and explore the characteristics of mutual coupling in CLA.

### 2.1. Data Model without Mutual Coupling

**Figure 1:**
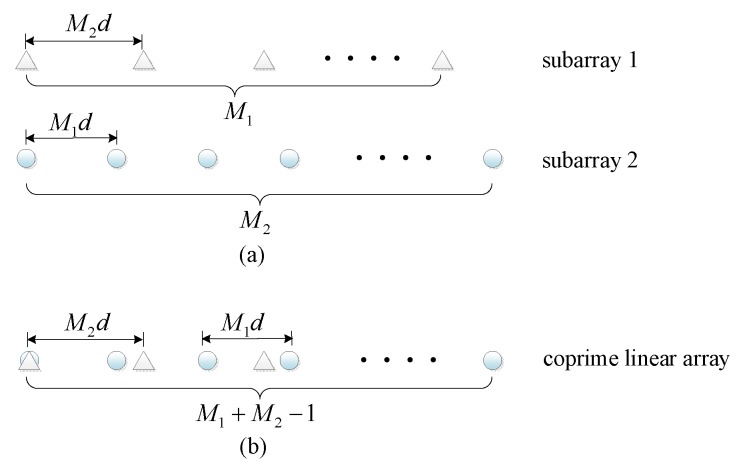
Array configurations. (**a**) the two subarrays; (**b**) the coprime linear array (CLA).

The CLA under study in this paper is composed of two uniform linear subarrays with $M_{1}$ and $M_{2}$ omnidirectional sensors, as illustrated in Figure 1. The inter-element spacing of the subarray 1 with *M*_1_ sensors is $d_{1}=M_{2}d$ while that of the subarray 2 with $M_{2}$ sensors is $d_{2}=M_{1}d$, where $M_{1}$ and $M_{2}$ are coprime integers,d=λ/2 and λ represents the wavelength. Without loss of generality, assume that $M_{1}<M_{2}$. The CLA has $M=M_{1}+M_{2}-1$ sensors in total and the location set of elements can be given by $L_{\mathrm{CLA}}=S_{\mathrm{CLA}}d$, where $S_{\mathrm{CLA}}$ is expressed as

(1) $$S_{\mathrm{CLA}}=\{m_{1}M_{2},\;m_{1}\in \langle 0,M_{1}-1\rangle \}\cup \{m_{2}M_{1},\;m_{2}\in \langle 0,M_{2}-1\rangle \}.$$

Assume that *K* far-field uncorrelated narrowband signals impinging on the CLA from distinct directions $\theta_{k}(k\in \langle 1,K\rangle )$. The received data of the CLA can be represented by

(2) $$\begin{array}{ll} x(t) & =\left[\begin{array}{c} A_{1}\\ A_{2} \end{array}\right]s(t)+\left[\begin{array}{c} n_{1}(t)\\ n_{2}(t) \end{array}\right]\\ & =As(t)+n(t) \end{array},$$

where $s(t)={\left[s_{1}(t),s_{2}(t),\cdots ,s_{K}(t)\right]}^{T}$ represents the signal vector, $n_{i}(t)$ is the white Gaussian noise, t∈〈1,L〉 and *L* is the number of snapshots. $A={\left[A_{1}^{T},A_{2}^{T}\right]}^{T}$ is the total steering matrix of the CLA and the corresponding steering vector is $a(\theta_{k})={\left[a_{1}^{T}(\theta_{k}),a_{2}^{T}(\theta_{k})\right]}^{T}$.$A_{i}=[a_{i}(\theta_{1}),a_{i}(\theta_{2}),\cdots ,a_{i}(\theta_{K})]$ (i=1,2) is the steering matrix of the *i*-th subarray, where $a_{i}(\theta_{k})={\left[1,e^{-j2\pi d_{i}\sin\theta_{k}/\lambda },\cdots ,e^{-j2\pi (M_{i}-1)d_{i}\sin\theta_{k}/\lambda }\right]}^{T}$. The total steering matrix of the CLA can be also constructed by $A_{c}=[a_{c}(\theta_{1}),a_{c}(\theta_{2}),\cdots ,a_{c}(\theta_{K})]$ after row exchange, where $a_{c}(\theta_{k})={\left[e^{-j\pi l_{1}\sin\theta_{k}},e^{-j\pi l_{2}\sin\theta_{k}},\cdots ,e^{-j\pi l_{M}\sin\theta_{k}}\right]}^{T}$ and $l_{m}\in S_{CLA}(m\in \langle 1,M\rangle )$. 

In particular, the CLA can be extracted from a filled ULA (FULA) with inter-element spacing d=λ/2 and $M_{F}=M_{1}(M_{2}-1)+1$ sensors. As a result, we have $A_{c}=G_{1}A_{F}$, where $A_{F}=[a_{F}(\theta_{1}),$ $a_{F}(\theta_{2}),\cdots ,a_{F}(\theta_{K})]$ denotes the steering matrix of the FULA and $a_{F}(\theta_{k})={\left[1,e^{-j\pi \sin\theta_{k}},\cdots ,e^{-j\pi (M_{F}-1)\sin\theta_{k}}\right]}^{T}$. $G_{1}$ is an $M\times M_{F}$ selection matrix composed of the $t_{m}-\mathrm{th}$$\left(t_{m}=l_{m}+1,\;m\in \langle 1,M\rangle \right)$ rows of $I_{M_{F}}$.

In practice, the covariance of the received signal x(t) is usually estimated by using *L* snapshots

(3) $$\hat{R}=\frac{1}{L}\sum_{t=1}^{L}x(t)x^{H}(t).\;$$

Performing eigenvalue decomposition of $\hat{R}$ and we have

(4) $$\hat{R}={\hat{U}}_{s}{\hat{\Lambda }}_{s}{\hat{U}}_{s}^{H}+{\hat{U}}_{n}{\hat{\Lambda }}_{n}{\hat{U}}_{n}^{H},\;$$

where ${\hat{\Lambda }}_{s}$ and ${\hat{\Lambda }}_{n}$ denotes the diagonal matrices composed of the *K* largest and the remaining eigenvalues of $\hat{R}$, respectively. ${\hat{U}}_{s}$ and ${\hat{U}}_{n}$ represent the signal subspace and noise subspace composed of the corresponding eigenvectors, respectively.

### 2.2. Data Model with Mutual Coupling

In the presence of mutual coupling, the model in Equation (2) should be reconstructed by

(5) $$\tilde{x}(t)=CAs(t)+n(t),\;$$

where C represents the mutual coupling matrix (MCM) and depends on practical factors, e.g., the type of antennas. For instance, the MCM of a linear dipole array with M elements can be written as [24,33,38,39]

(6) $$C=(Z_{A}+Z_{L}){\left(Z+Z_{L}I_{M}\right)}^{-1},\;$$

where $Z_{A}$ and $Z_{L}$ denote the antenna/load impedance, respectively. Z is the mutual impedance matrix and can be expressed as

(7) $$\{\begin{array}{cc} Z=\frac{\eta_{0}}{4\pi }(0.5772+\ln(2\beta l)-Ci(2\beta l)+jSi(2\beta l)), & p=q\\ Z=\frac{\eta_{0}}{4\pi }({ℜ}_{p,q}+j{ℵ}_{p,q}), & p\neq q \end{array},\;$$

where β=2π/λ, *l* denotes the length of dipole antennas and

(8) $$\begin{array}{ll} {ℜ}_{p,q} & =\sin(\beta l)(-Si(u_{0})+Si(v_{0})+2Si(u_{1})-2Si(v_{1}))\\ & +cos(\beta l)(Ci(u_{0})+Ci(v_{0})-2Ci(u_{1})-2Ci(v_{1})+2Ci(\beta d_{p,q}))\\ & -(2Ci(u_{1})+2Ci(v_{1})-4Ci(\beta d_{p,q})) \end{array},$$

(9) $$\begin{array}{ll} {ℵ}_{p,q} & =\sin(\beta l)(-Ci(u_{0})+Ci(v_{0})+2Ci(u_{1})-2Ci(v_{1}))\\ & +cos(\beta l)(-Si(u_{0})-Si(v_{0})+2Si(u_{1})+2Si(v_{1})-2Si(\beta d_{p,q}))\\ & +(2Si(u_{1})+2Si(v_{1})-4Si(\beta d_{p,q})) \end{array},$$

where $d_{p,q}$ is the distance between the *p*-th element and the *q*-th element, $u_{0}=\beta (\sqrt{d_{p,q}^{2}+l^{2}}-l)$, $u_{1}=\beta (\sqrt{d_{p,q}^{2}+0.25l^{2}}-0.5l)$,$v_{0}=\beta (\sqrt{d_{p,q}^{2}+l^{2}}+l)$ and $v_{1}=\beta (\sqrt{d_{p,q}^{2}+0.25l^{2}}+0.5l)$. Si(u) and Ci(u) are defined as

(10) $$Si(u)=\int_{\infty }^{u}\frac{\sin x}{x}dx,\;Ci(u)=\int_{\infty }^{u}\frac{\cos x}{x}dx.$$

**Figure 2:**
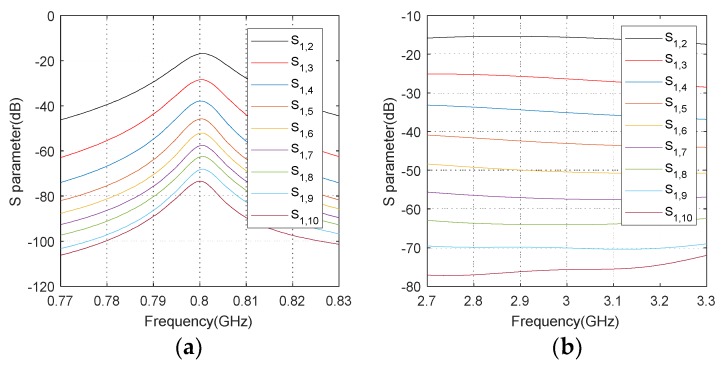
*S*-parameter characteristics of two kinds of antenna array. (**a**) Microstrip array; (**b**) Dipole array.

In order to facilitate the theoretical study of parameter estimation, a series of simplified models of mutual coupling are established based on certain assumptions [24,25,26,27,28,29,30,31,32,33,34,35,36,37,38,39,40]. In this paper, we assume that $c_{p,q}$ can be characterized by [24,33,38,39]

(11) $$c_{p,q}=\{\begin{array}{cc} 0, & |d_{p}-d_{q}|\geq B\\ c_{\left|d_{p}-d_{q}\right|}, & |d_{p}-d_{q}|<B \end{array},$$

where $d_{p},d_{q}\in S_{\mathrm{CLA}}$, $c_{p,q}$ represents the coupling effect between the *q*-th sensor and the *p*-th sensor in CLA. $c_{b}=c_{\left|d_{p}-d_{q}\right|}$ are defined as the MCCs which satisfy $1=c_{0}>|c_{1}|>|c_{2}|>\cdots >|c_{B}|$ =0. The threshold of mutual coupling range B=3 and B=5 are commonly selected assumptions in the existing work [32,40,41]. Specifically, as the mutual coupling depends on practical factors, the value of *B* is generally determined by the actual scenario. To explain it more intuitively and provide a reliable foundation for the proposed scheme, we provide the *S*-parameter characteristics of two kinds of arrays below as an example. In practical applications, *S*-parameters are typically used to characterize the mutual coupling and $S_{p,q}$ captures the coupling degree between the *p*-th element and the *q*-th element [42]. Figure 2 exhibits the *S*-parameter characteristics from an electromagnetic simulation software, i.e., HFSS, where two ten-element ULAs composed of microstrip antennas and dipole antennas are studied. The operating frequencies corresponding to the two arrays are 0.8 GHz and 2.95 GHz. It is generally believed that the mutual coupling is relatively weak when $S_{p,q}$ is lower than -40dB [43,44], and by assuming that the mutual coupling effect can be ignored when $S_{p,q}<55\mathrm{dB}$, the value of *B* in the above two arrays can take 5 and 6, respectively.

**Figure 3:**
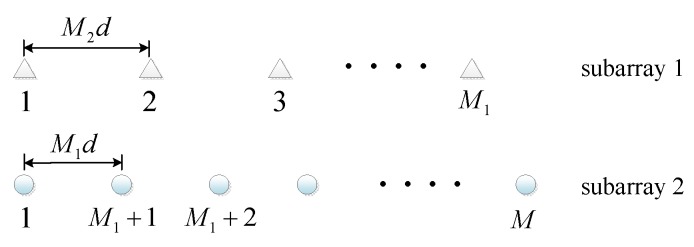
The labels of the sensors in CLA.

According to the labels of the sensors in Figure 3, C can be partitioned by

(12) $$\begin{array}{ll} C & =[\begin{array}{ccccccccc} 1 & c_{1,2} & c_{1,3} & \cdots & c_{1,M_{1}} & c_{1,M_{1}+1} & c_{1,M_{1}+2} & \cdots & c_{1,M}\\ c_{2,1} & 1 & c_{2,3} & \ddots & c_{2,M_{1}} & c_{2,M_{1}+1} & c_{2,M_{1}+2} & \cdots & c_{2,M}\\ c_{3,1} & c_{3,2} & 1 & \cdots & c_{3,M_{1}} & & \vdots & & \vdots \\ \vdots & \vdots & \vdots & \ddots & \vdots & \vdots & \cdots & \ddots & \vdots \\ c_{M_{1},1} & c_{M_{1},2} & c_{M_{1},3} & \cdots & 1 & c_{M_{1},M_{1}+1} & c_{M_{1},M_{1}+2} & \cdots & c_{M_{1},M}\\ c_{M_{1}+1,1} & c_{M_{1}+1,2} & & \cdots & c_{M_{1}+1,M_{1}} & 1 & c_{M_{1}+1,M_{1}+2} & \cdots & c_{M_{1}+1,M}\\ c_{M_{1}+2,1} & c_{M_{1}+2,2} & \cdots & \vdots & c_{M_{1}+2,M_{1}} & c_{M_{1}+2,M_{1}+1} & 1 & \vdots & c_{M_{1}+2,M}\\ \vdots & \vdots & & \ddots & \vdots & \vdots & \cdots & \ddots & \vdots \\ c_{M,1} & c_{M,2} & \cdots & \cdots & c_{M,M_{1}} & c_{M,M_{1}+1} & c_{M,M_{1}+2} & \cdots & 1 \end{array}],\\ & =\left[\begin{array}{cc} D_{1} & B_{1}\\ B_{2} & D_{2} \end{array}\right] \end{array}$$

where $D_{i}\in {\mathbb{C}}^{M_{i}\times M_{i}}$ denotes the MCM in the *i*-th (i=1,2) subarray while $B_{1}\in {\mathbb{C}}^{M_{1}\times M_{2}}$ and $B_{2}\in {\mathbb{C}}^{M_{2}\times M_{1}}$ capture the coupling effect between the two subarrays. In particular, $C=C^{T}$.

Since the inter-element spacings in the two subarrays of CLA are large, we can conclude that the mutual coupling in CLA is mainly caused by the interaction between the two subarrays. It is noteworthy that most of the elements in $D_{1}$ and $D_{2}$ are zero, and especially when $M_{i}\geq B$, no mutual coupling exists in the *j*-th subarray (i=1,2;j=3−i), which motivates us to propose the array switching scheme to mitigate the mutual coupling in CLA by exploiting the inter-element spacings of the two subarrays. 

## 3. The Proposed Parameter Estimation Scheme

In this section, we introduce the proposed scheme to deal with mutual coupling for improved parameter estimation performance through the array switching strategy. 

**Figure 4:**
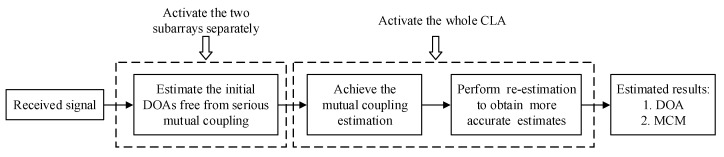
The implementation flow of the proposed scheme.

Figure 4 presents the implementation flow of the proposed scheme. Based on the inherent sparse structural characteristic of CLA, we first estimate the initial DOAs by separately activating the two subarrays, which can avoid the mutual coupling resulting from the interlaced subarrays. Then the two interactive subarrays receive signals at the same time and the MCCs of the total CLA can be estimated by utilizing the initial DOA estimates. Furthermore, re-estimation is performed by an iteration procedure based on the estimated MCM to refine parameter estimates. 

### 3.1. Initial DOA Estimation

To begin with, the two subarrays are separately activated to collect signals $X_{1}^{ini}$ and $X_{2}^{ini}$, which are inherently less susceptible to mutual coupling benefiting from the large inter-element spacings. As a result, we directly apply the uncalibrated polynomial rooting technique to the two subarrays respectively, then the initial unambiguous DOAs can be determined by utilizing the coprime property [17].

In this situation, the MCMs of the two subarrays are assumed to be identity matrices and then the MUSIC spectrum function of the *i*-th subarray can be represented by [17]

(13) $$f_{i}(\theta )=\frac{1}{a_{i}^{H}(\theta )U_{i,n}^{ini}{\left(U_{i,n}^{ini}\right)}^{H}a_{i}^{H}(\theta )},\;$$

where $U_{i,n}^{ini}$ is the noise subspace corresponding to $X_{i}^{ini}$. Let $z_{i}=e^{-j2\pi d_{i}\sin\theta /\lambda }$, we have $a_{i}(\theta )=$ $a(z_{i})={\left[1,z_{i},\cdots ,z_{i}^{M_{i}-1}\right]}^{T}$ and the DOA estimation is transformed into solving the roots of the polynomial [11]

(14) $$a_{i}^{T}(z^{-1})U_{i,n}^{ini}{\left(U_{i,n}^{ini}\right)}^{H}a_{i}(z)=0,\;$$

Consequently, the initial ambiguous DOA estimates can be achieved by

(15) $$\sin({\hat{\theta }}_{i,k}^{ini})=-angle({\hat{z}}_{i,k})\lambda /2\pi d_{i}\;(k\in \langle 1,K\rangle ),\;$$

The phase ambiguity, which stems from the inter-element spacing larger than λ/2, can be eliminated based on the coprime property [17]. As a result, the true estimates ${\hat{\theta }}_{k}^{ini}$ can be uniquely determined from the *K* pairs of closest estimates of the two subarrays. Once the DOA estimates ${\hat{\theta }}_{k}^{ini}$ free from severe mutual coupling is obtained, we can further use it to estimate the MCCs by receiving the signal X of the total CLA.

### 3.2. Mutual Coupling Estimation

Herein, we first reconstruct the contaminated steering vector of the whole CLA to decouple the DOA and mutual coupling. Subsequently, a quadratic optimization problem is established to achieve the mutual coupling estimation. Finally, refined DOA estimates can be obtained via an iteration procedure.

Due to the banded symmetric Toeplitz characteristic of the MCM in ULA [24], the contaminated steering vector of the FULA can be transformed into $C_{F}a_{F}(\theta )=T(a_{F}(\theta ))c_{F}$, where $C_{F}\in {\mathbb{C}}^{M_{F}\times M_{F}}$ and $c_{F}={\left[1,c_{1},c_{2},\ldots ,c_{B-2}\right]}^{T}$ are the MCM and mutual coupling vector of the FULA, respectively. The steering vector transformation matrix $T(a_{F}(\theta ))\in {\mathbb{C}}^{M_{F}\times B}$ can be obtained by

(16) $$T(a_{F}(\theta ))=T_{1}(a_{F}(\theta ))+T_{2}(a_{F}(\theta )),\;$$

(17) $${\left[T_{1}(a_{F}(\theta ))\right]}_{p,q}=\{\begin{array}{cc} {\left[a_{F}(\theta )\right]}_{p+q-1}, & p+q\leq M_{F}+1\\ 0, & \mathrm{otherwise} \end{array},$$

(18) $${\left[T_{2}(a_{F}(\theta ))\right]}_{p,q}=\{\begin{array}{cc} {\left[a_{F}(\theta )\right]}_{p-q-1}, & p\geq q\geq 2\\ 0, & \mathrm{otherwise} \end{array}.$$

Due to the nonuniform array structure, the MCM of CLA no longer has such special banded symmetric Toeplitz form. As a result, the reconstruction of the steering vector in ULA cannot be directly applied for further decoupling. To address this, we utilize two matrices $G_{0}$ and $G_{1}$ derived from the relation between FULA and CLA to reconstruct the contaminated steering vector of CLA as

(19) $$\begin{array}{ll} \tilde{a}(\theta ) & =Ca(\theta )\\ & =G_{1}C_{F}(G_{0}a_{F}(\theta ))\\ & =G_{1}T({a^{\prime }}_{F}(\theta ))c_{F}\\ & =H(\theta )c_{F} \end{array},$$

where ${a^{\prime }}_{F}(\theta )=G_{0}a_{F}(\theta )$ and $G_{0}\in {\mathbb{C}}^{M_{F}\times 1}$ is a column vector whose $t_{m}-\mathrm{th}$$\left(t_{m}=d_{m}+1,\;m\in \langle 1,M\rangle \right)$ elements are 1 and the rest entries are 0. It is noteworthy that this reconstruction method of the contaminated steering vector is applicable to other nonuniform linear arrays.

Based on the transformation in Equation (19), the mutual coupling estimation can be achieved from the following quadratic optimization problem

(20) $$\underset{c_{F}}{\min}c_{F}{}^{H}H^{H}({\hat{\theta }}^{ini})U_{n}U_{n}^{H}H({\hat{\theta }}^{ini})c_{F},\;s.t.\;e^{H}c_{F}=1,\;$$

where $U_{n}$ is the noise subspace of the signal X received by the whole CLA and $e_{1}={}^{T}\in {\mathbb{R}}^{B\times 1}$. Construct the Lagrange function as

(21) $$L(\theta ,c_{F})=c_{F}{}^{H}Q(\theta )c_{F}-\varepsilon (e_{1}{}^{H}c_{F}-1),\;$$

where $Q(\theta )=H^{H}(\theta )U_{n}U_{n}^{H}H(\theta )$, ε stands for a Lagrange multiplier. By taking the partial derivative of $L(\theta ,c_{F})$, i.e., $\partial L(\theta ,c_{F})/\partial c_{F}=2Q(\theta )c_{F}-\varepsilon e_{1}=0$, we can obtain the estimates of $c_{F}$ by

(22) $${\hat{c}}_{F,k}=\frac{Q^{-1}({\hat{\theta }}_{k}^{ini})e_{1}}{e_{1}^{H}Q^{-1}({\hat{\theta }}_{k}^{ini})e_{1}},\;$$

(23) $${\hat{c}}_{F}=\frac{1}{K}\sum_{k=1}^{K}{\hat{c}}_{F,k}.\;$$

Subsequently, the MCM of CLA can be constructed by

(24) $$\hat{C}=G_{1}{\hat{C}}_{F}G_{1}^{T},\;$$

where ${\hat{C}}_{F}=\mathrm{Toeplitz}\text{\{}{\hat{c}}_{F},0_{1\times (M_{F}-B)}\text{\}}$.

### 3.3. Iteration Procedure for Refined Estimation

Since there exists a certain deviation between the spectral function in Equation (13) and the actual one contaminated by mutual coupling, the performance of the initial estimation is still potentially degraded. For further improvement in estimation performance, an iteration approach based on the initial MCM estimate $\hat{C}$ is designed in this part. The detailed steps are outlined in Table 1. 

Note that the conventional iteration-based mutual coupling calibration algorithm [30,36] usually sets $C^{\left(0\right)}=I$, whereas the convergence speed and estimation accuracy heavily depends on $C^{\left(0\right)}$. The proposed scheme can provide well-performed initial DOA estimates which contributes to the estimation of MCM, thus it requires few iterations and can gain superior estimation performance simultaneously.

### 3.4. Procedure of the Proposed Scheme 

The detailed steps for implementation of the proposed scheme is summarized below.

1. Activate the two subarrays of CLA separately and compute $R_{i}^{ini}$ with $X_{i}^{ini}$ to obtain $U_{i,n}^{ini}$ (i=1,2).

2. Employ the polynomial rooting according to Equations (14)–(15) and distinguish the unambiguous angles ${\hat{\theta }}_{k}^{ini}$ based on the coprime property.

3. Activate the whole CLA and calculate R with the received signal X to obtain $U_{n}^{}$.

4. Construct Q(θ) and achieve the mutual coupling estimation according to Equations (20)–(24).

5. Perform the re-estimation to further improve the estimation accuracy.

**Remark** **1.** *It is assumed that the DOAs and statistical characteristics of the signals are constant during the array switching process.*

**Remark** **2.** *Based on the steering vector reconstruction in Equation (19), the well-known rank-reduction (RARE) estimator [33,34] can be extended from ULA to CLA to achieve DOA and mutual coupling estimation, which is provided in the comparison and termed as RARE-based calibration in this paper.*

## 4. Performance Analysis

### 4.1. Complexity Analysis

The computational complexity of the proposed scheme is analyzed in this part and it is represented by the number of complex multiplications. In the process of initial DOA estimation, calculating the covariance $R_{i}^{ini}(i=1,2)$ with *L* snapshots requires $O(L(M_{1}^{2}+M_{2}^{2}))$ and performing eigenvalue decomposition costs $O(M_{1}^{3}+M_{2}^{3})$. Constructing the polynomial needs $O(2M_{1}(M_{1}-K)$ $+M_{1}+2M_{2}(M_{2}-K)+M_{2})$ and the complexity of root-finding operation is $O({\left(M_{1}-1\right)}^{3}+{\left(M_{2}-1\right)}^{3})$. As for the mutual coupling estimation, constructing $Q(\theta^{ini})$ takes $O(M^{3}+LM^{2}+B^{2}M+MM_{F}B+$ 2M(M−K)B) operations and calculating $Q^{-1}(\theta^{ini})$ costs $O(B^{3})$. The complexity of the recovery of MCM is $O(MM_{F}^{2}+M^{2}M_{F}^{})$. The complexity calculation of the iteration process is similar to that of the previous part. Table 2 lists the complexity of the proposed scheme in steps, where $n_{iter}$ stands for the iteration times.

**Figure 5:**
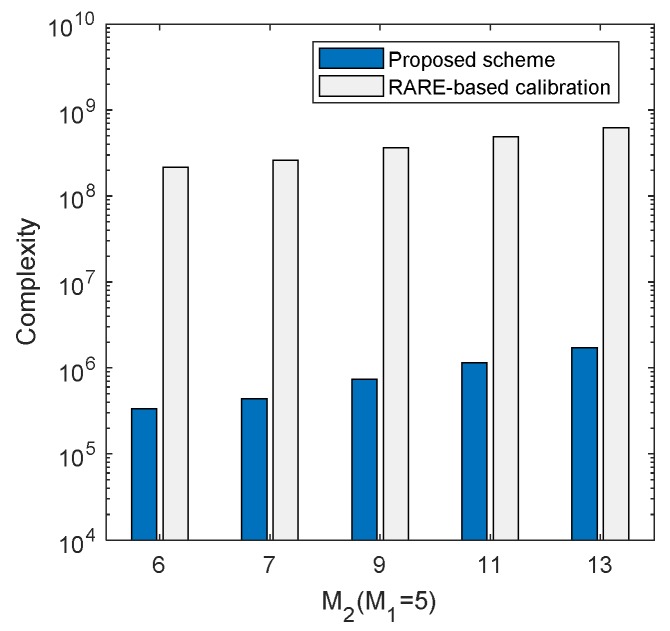
Complexities of different schemes versus different $M_{2}$.

**Figure 6:**
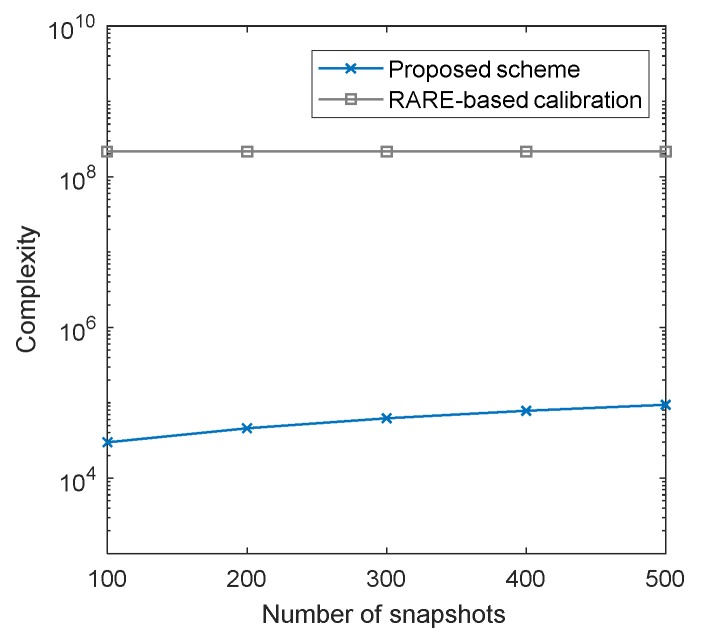
Complexities of different schemes versus different snapshots.

In addition, we compare the complexity of the proposed scheme with the RARE-based calibration scheme. Table 3 gives the total complexities of the two schemes, where $n_{1}=180/d_{s}$ is the peak search times and $d_{s}$ is the searching grid. The complexity comparisons of the two schemes are illustrated in Figure 5 and Figure 6, where B=6, $d_{s}=0.001^{\circ }$ and $n_{iter}=3$. Figure 5 exhibits the complexity comparison versus $M_{2}$, where $M_{1}=5$, K=3 and L=500, while the comparison versus snapshots is presented in Figure 6, where $M_{1}=5$, $M_{2}=6$ and K=3. It can be seen clearly in Figure 5 and Figure 6 that the proposed scheme requires significantly lower computational cost than the RARE-based calibration method, which is attractive for online calibration.

### 4.2. Mutual Coupling Analysis

In this part, we employ the coupling leakage [38] to measure the inhibition of the proposed scheme on mutual coupling which is defined as $\gamma ={\left\|C-diag(C)\right\|}_{F}/{\left\|C\right\|}_{F}$. Conceptually, the smaller γ, the weaker the mutual coupling is.

Table 4 exhibits the coupling leakage comparison of the ULA, the general CLA [12] and the CLA based on array switching with the number of sensors, where the MCCs are provided as in Equation (11) with $c_{1}=0.3e^{j\pi /3}$ and $c_{b}=c_{1}e^{-j\pi (b-1)/8}/b$ for 2≤b<B. As depicted in Table 4, compared with the general CLA, the mutual coupling effect is significantly alleviated in the CLA by exploiting the proposed array switching strategy, as the severe mutual coupling caused by the interleaved subarrays is avoided.

### 4.3. Cramer-Rao Bound

To provide a benchmark of parameter estimation performance, the Cramer-Rao Bound (CRB) [24] with CLA under unknown mutual coupling is derived. Define the vector of unknown parameters as

(25) $$p^{T}=[\theta_{1},\theta_{2},\cdots ,\theta_{K},\rho_{1},\rho_{2},\cdots ,\rho_{B-1},\eta_{1},\eta_{2},\cdots ,\eta_{B-1}],$$

where $\theta_{k}(k\in \langle 1,K\rangle )$ represents the DOAs of the signals. $\rho_{b}$ and $\eta_{b}(b\in \langle 1,B-1\rangle )$ are the real and imaginary parts of the MCCs. The CRB corresponding to p can be determined by

(26) $$\mathrm{CRB}=diag(F^{-1}),\;$$

where F is the Fisher information matrix (FIM) and can be represented by

(27) $$F=\left[\begin{array}{ccc} F_{\theta \theta } & F_{\theta \rho } & F_{\theta \eta }\\ F_{\rho \theta } & F_{\rho \rho } & F_{\rho \eta }\\ F_{\eta \theta } & F_{\eta \rho } & F_{\eta \eta } \end{array}\right].$$

The (*u*,*v*)-the element of the FIM can be specified as

(28) Fu,v=L×tr[R−1∂R∂puR−1∂R∂pv]=2L×Re{tr[DuRsA˜HR−1A˜RsDvR−1]+tr[DuRsA˜HR−1DvHRsA˜HR−1]}

where $\tilde{A}=CA$, $D_{u}=\partial \tilde{A}/\partial p_{u}$ and $D_{v}=\partial \tilde{A}/\partial p_{v}$.

### 4.4. Advantages

The proposed scheme for CLA against mutual coupling has the following advantages:

(1) The proposed scheme can be employed as an online calibration technique, which requires no extra auxiliary sources or auxiliary sensors.
(2) The proposed scheme can significantly alleviate the mutual coupling by exploiting the structural characteristics of CLA. In particular, it outperforms the RARE-based and iterative calibration methods in parameter estimation, which is illustrated in Section 5.
(3) The proposed scheme is computationally efficient since no spectral search is required, which is attractive in practical applications.

## 5. Simulation Results

In this section, extensive simulations are carried out to corroborate the superiority of the proposed scheme. The root mean square error (RMSE) with 1000 Monte Carlo trials is defined as

(29) $$\mathrm{RMSE}=\sqrt{\frac{1}{1000K}\sum_{\nu =1}^{1000}\sum_{k=1}^{K}{\left(\theta_{k}-{\hat{\theta }}_{k,\nu }\right)}^{2}},\;$$

where $\theta_{k}$ and ${\hat{\theta }}_{k,\nu }$ denote the theoretical DOA and the estimate for the *v*-th trial of the *k*-th signal, respectively.

### 5.1. Verification of the Parameter Estimation

**Figure 7:**
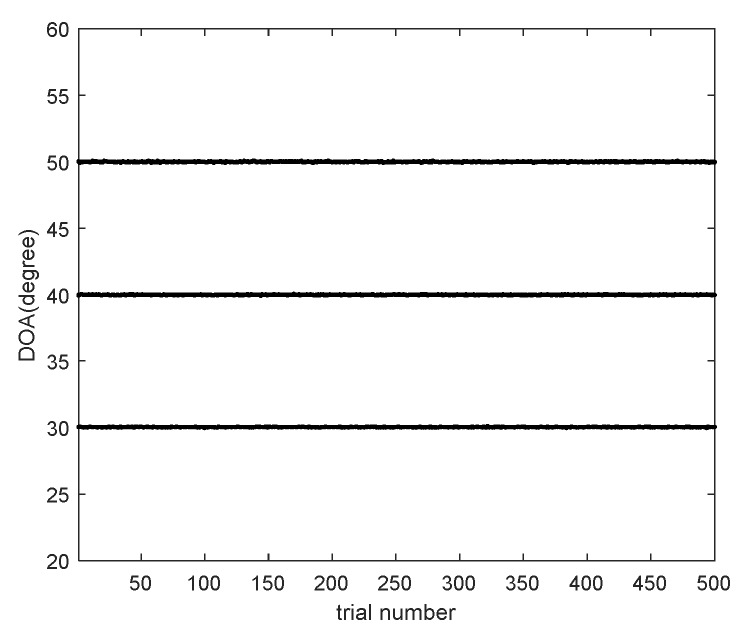
Direction of arrival (DOA) estimation results of the proposed scheme.

**Figure 8:**
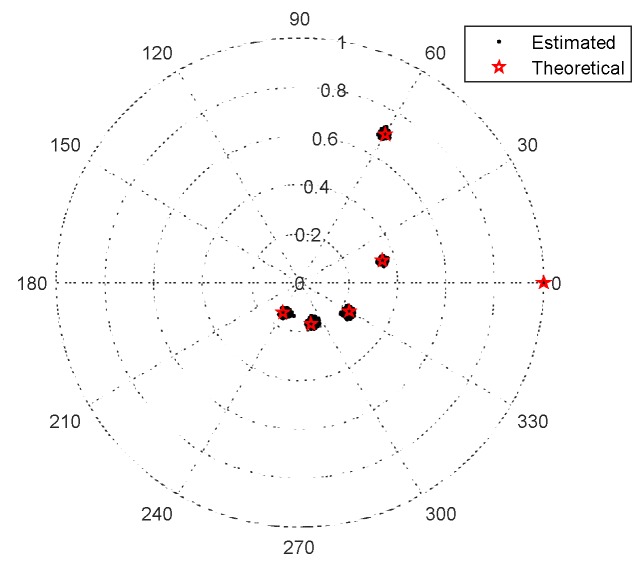
Mutual coupling coefficients (MCCs) estimation results of the proposed scheme.

We provide the DOA estimation results of the proposed scheme in Figure 7, where $M_{1}=5$, $M_{2}=6$, SNR=10dB, L=500 and B=6. Consider K=3 narrowband uncorrelated signals with $\left[\theta_{1},\theta_{2},\theta_{3}]=[30^{\circ },40^{\circ },50^{\circ }\right]$. The MCCs are set as $c_{1}=0.7e^{j\pi /3}$ and $c_{b}=c_{1}e^{-j\pi (b-1)/4}/b$, 2≤b<B. It is illustrated in Figure 7 that all signals can be resolved correctly. In addition, Figure 8 presents the estimation results of MCCs in polar coordinates and Table 5 lists the mean values and estimation biases of the estimated MCCs, where the estimation bias is defined as $\varepsilon_{r}=\sum_{\nu =1}^{1000}{\left\|c_{b}-{\hat{c}}_{b,\nu }\right\|}_{F}$ $/{\left\|c_{b}\right\|}_{F}/1000$, $c_{b}$ and ${\hat{c}}_{b,\nu }$ represent the theoretical value and the estimate for the *v*-th trial of the *b*-th MCC, respectively. The results demonstrate that the proposed scheme can obtain well-performed MCC estimates, which, in turn, contribute to the refined DOA estimates.

### 5.2. RMSE Performance of Different Schemes

In this example, we compare the RMSE results of different schemes, including the proposed scheme without iterations (marked as ‘‘proposed scheme-initial’’), the proposed scheme with iterations (marked as ‘‘proposed scheme-refined’’), the mutual coupling calibration schemes (the RARE-based, auxiliary source-based [35] and iterative calibration [36] methods), the uncalibrated root-MUSIC algorithm with unknown mutual coupling, where K=2, $\left[\theta_{1},\theta_{2}]=[30^{\circ },40^{\circ }\right]$, $M_{1}=6$, $M_{2}=7$, B=8, $c_{1}=0.7e^{j\pi /3}$, $c_{b}=c_{1}e^{-j\pi (b-1)/8}/b$ and the searching grid of spectral search is set to $d_{s}=0.0001^{\circ }$. The estimation performance of the root-MUSIC [45] in the absence of mutual coupling and the CRB are also plotted for reference.

**Figure 9:**
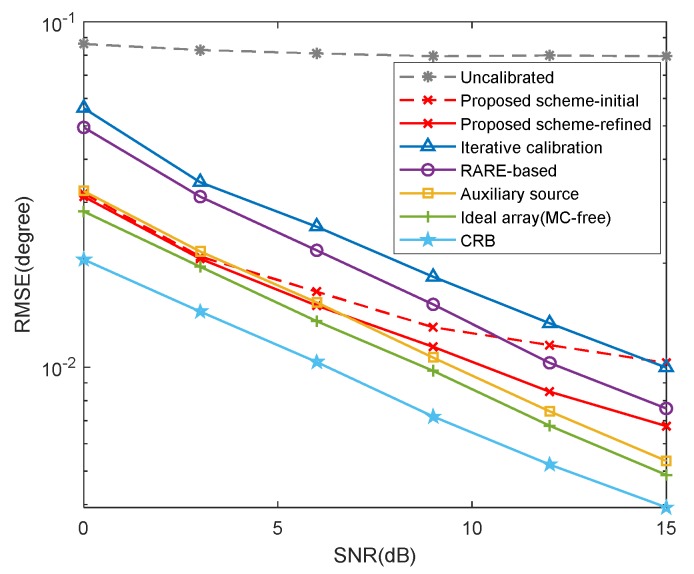
Root mean square error (RMSE) of different schemes versus signal-to-noise ratio (SNR) (*L*=500).

Figure 9 exhibits the RMSE results versus signal-to-noise ratio (SNR), where L=500. It is indicated explicitly that the uncalibrated root-MUSIC method [45] performs the worst and achieves little improvement in performance with the increase of SNR, which signifies that even though the mutual coupling effect in CLA is relatively weak, it still degrades the performance. By comparison, since the subarrays are less susceptible to mutual coupling benefiting from the large inter-element spacing, the proposed scheme can obtain well-performed initial DOA estimates, which is even superior to the RARE-based algorithm at low SNRs and contributes to the estimation of MCM. As the mutual coupling is not compensated or calibrated in the initial estimation process, the estimation performance of the initial estimates is inevitably restricted. By comparison, it is observed that refined DOA estimates, especially at high SNRs, can be achieved via an iteration procedure by utilizing the MCM estimates. In particular, the proposed scheme can obtain similar DOA estimates to the method with auxiliary source. The estimation performance of the auxiliary source-based method generally improves with the increase of the number of auxiliary sources to some extent, whereas it is exceedingly difficult to implement in practical systems.

**Figure 10:**
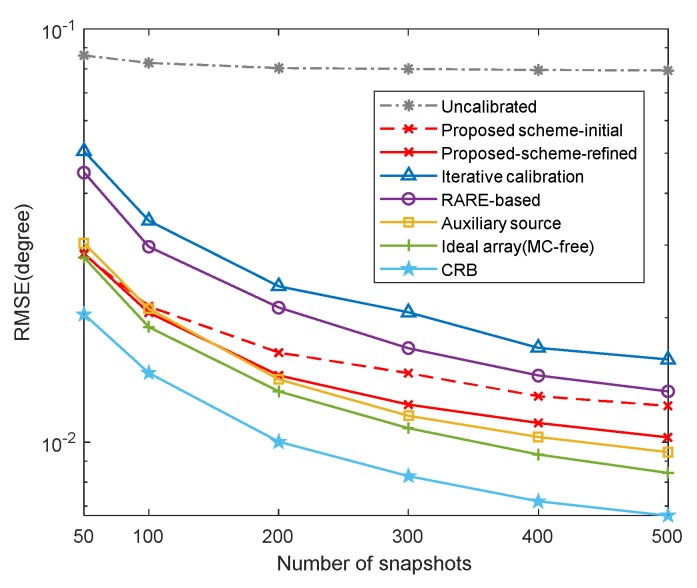
RMSE of different schemes versus snapshots (SNR=10dB).

Meanwhile, the RMSE comparison in terms of snapshots is captured in Figure 10, where SNR=10dB. It is illustrated that the RMSE results improve with the number of snapshots increasing due to the more accurate estimated covariance matrix. We can also conclude from Figure 10 that the proposed scheme can provide as good DOA estimation performance comparable to the calibration methods, indicating that the array switching strategy can effectively alleviate the mutual coupling effect.

### 5.3. RMSE Performance of Different Mutual Coupling

**Figure 11:**
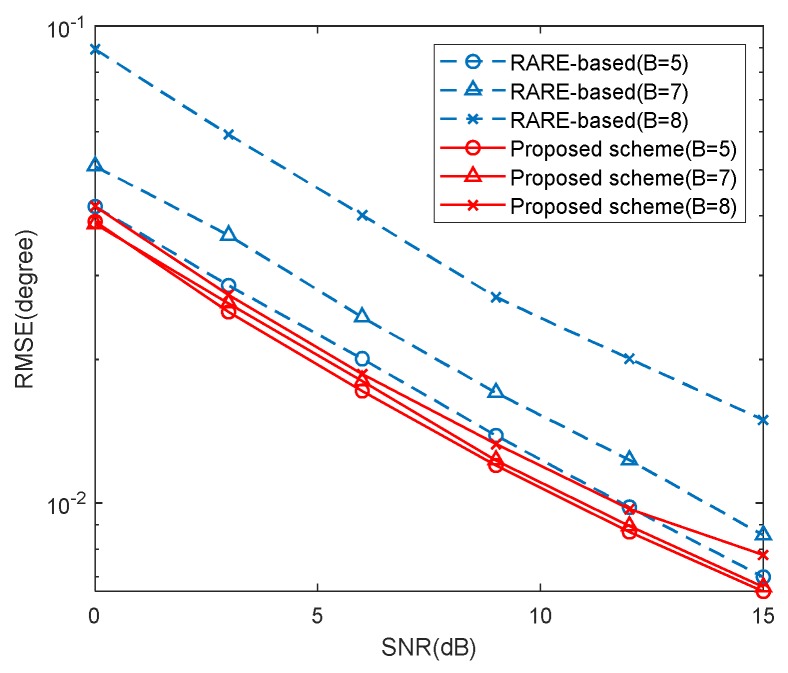
RMSE with different mutual coupling versus SNR (*L*=500).

**Figure 12:**
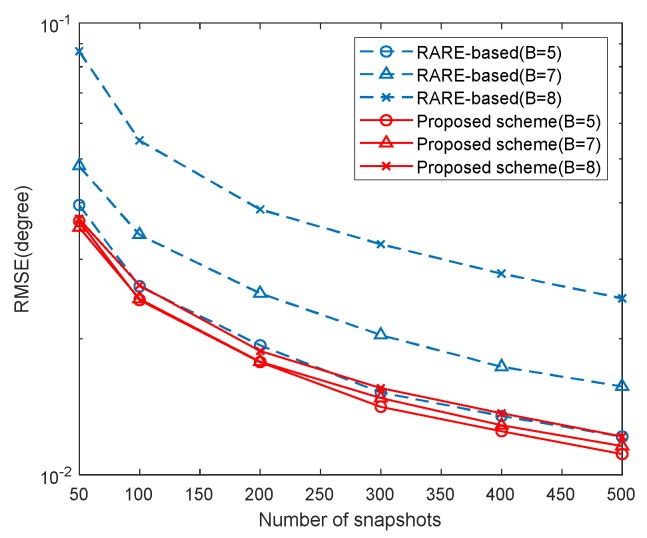
RMSE with different mutual coupling versus snapshots (SNR=10dB).

In this example, we provide the RMSE results of the proposed scheme with different *B* versus SNR and snapshots in Figure 11 and Figure 12, where K=2, $\left[\theta_{1},\theta_{2}]=[30^{\circ },40^{\circ }\right]$, $M_{1}=5$, $M_{2}=7$ and the MCCs are the same as those in Section 5.2. In the two examples, we set L=500 and SNR=10dB, respectively. The RMSE results of the RARE-based calibration are also given for comparison. It is indicated by Figure 11 and Figure 12 that the DOA estimation performance of the two methods deteriorates with strong mutual coupling effect. Besides, the proposed scheme yields more accurate DOA estimates than the RARE-based calibration in all three cases and has better robustness of the range of mutual coupling.

## 6. Conclusions

In this paper, we have presented an array switching strategy for DOA estimation with CLA in the presence of mutual coupling. Due to the nonuniform array configuration of CLA, the conventional calibration methods for ULAs become difficult to apply. Motivated by the sparse arrangement and the characteristics of mutual coupling in CLA, we design an array switching-based scheme to achieve the DOA estimation against mutual coupling. In the proposed scheme, the two subarrays are separately activated first to collect the signals and estimate the initial DOAs, then the total CLA is used to achieve the mutual coupling estimation and further re-estimate for refined estimates. As a consequence, the well-performed DOA estimates free from the severe mutual coupling effect can be obtained. Numerical simulations corroborate the superiority of the proposed scheme for CLA with regard to computational complexity, mutual coupling and parameter estimation performance. Considering that the mutual coupling is more complicated in practice, the calibration technique with modified coupling model, e.g., direction-dependent model, based on engineering applications will constitute our future work. 

## Figures and Tables

**Table 1:** Steps of the iteration procedure.

**Procedure:** Iterations for refined estimates
**Step 1: Initialization:** Initialize iter=0 and $C^{\left(0\right)}=\hat{C}$. **Step 2: DOA Estimation:** Obtain the DOA estimates $\theta^{\left(iter\right)}$ by solving the roots of polynomial $f^{\left(iter\right)}(z)=a_{}^{T}(z^{-1}){\left(C^{\left(iter\right)}\right)}^{H}U_{n}^{}U_{n}^{H}(C^{\left(iter\right)}a(z))=0$, then let iter=iter+1. **Step 3: Mutual Coupling Estimation:** Calculate $c_{F}^{\left(iter\right)}=Q^{-1}(\theta^{\left(iter\right)})e_{1}/(e_{1}^{H}Q^{-1}(\theta^{\left(iter\right)})e_{1})$ and further obtain $C^{\left(iter\right)}=G_{1}C_{F}^{\left(iter\right)}G_{1}^{T}$. **Step 4: Convergence Determination:** Go to step 2 until ${\left\|C^{\left(iter\right)}A^{\left(iter-1\right)}-C^{\left(iter-1\right)}A^{\left(iter-2\right)}\right\|}^{2}<\delta$, where δ is a given threshold, such as $10^{-2}$.

**Table 2:** Complexity of the proposed scheme.

Step	Computational complexity
Initial DOA Estimation	$O(L(M_{1}^{2}+M_{2}^{2})+M_{1}^{3}+M_{2}^{3}+2M_{1}(M_{1}-K)+2M_{2}(M_{2}-K)$ $+M_{1}+M_{2}+{\left(M_{1}-1\right)}^{3}+{\left(M_{2}-1\right)}^{3})$
Mutual Coupling Estimation	$O(M^{3}+(L+M_{F}^{})M^{2}+M(B^{2}+M_{F}^{2}+M_{F}B)+2M(M-K)B+B^{3})$
Iteration Process	$O(n_{iter}(2M(M-K)+M+{\left(M_{F}-1\right)}^{3})+(n_{iter}-1)(M_{F}^{}M^{2}$ $+M(B^{2}+M_{F}^{2}+M_{F}B)+2M(M-K)B+B^{3}))$
Total	$O(L(M_{1}^{2}+M_{2}^{2}+M^{2})+M_{1}^{3}+M_{2}^{3}+M^{3}+{\left(M_{1}-1\right)}^{3}+{\left(M_{2}-1\right)}^{3}$ $+2M_{1}(M_{1}-K)+2M_{2}(M_{2}-K)+M_{1}+M_{2}+n_{iter}(2M(M-K)(1+B)$ $+M+{\left(M_{F}-1\right)}^{3}+M_{F}^{}M^{2}+M(B^{2}+M_{F}^{2}+M_{F}B)+B^{3})$

**Table 3:** Complexities of different schemes.

Scheme	Computational complexity
Proposed	$O(L(M_{1}^{2}+M_{2}^{2}+M^{2})+M_{1}^{3}+M_{2}^{3}+M^{3}+{\left(M_{1}-1\right)}^{3}+{\left(M_{2}-1\right)}^{3}$ $+2M_{1}(M_{1}-K)+2M_{2}(M_{2}-K)+M_{1}+M_{2}+n_{iter}(2M(M-K)(1+B)$ $+M+{\left(M_{F}-1\right)}^{3}+M_{F}^{}M^{2}+M(B^{2}+M_{F}^{2}+M_{F}B)+B^{3})$
RARE-based calibration	$O(2LM^{2}+2M^{3}+n_{1}(2MM_{F}B+2M(M-K)B+MB^{2})+MM_{F}B$ $+M^{2}B+2M(M-K)B+B^{3}+MM_{F}^{2}+M^{2}M_{F}^{})$

**Table 4:** Comparison of coupling leakage γ among uniform linear arrays (ULA) and CLA in two cases.

Condition	ULA	CLA (General)	CLA (Switching-Based)
$M_{1}=5,M_{2}=6,M=10,B=5$	0.6177	0.3530	0
$M_{1}=5,M_{2}=6,M=10,B=10$	0.6251	0.3741	0.1156
$M_{1}=8,M_{2}=13,M=20,B=5$	0.6312	0.2578	0
$M_{1}=8,M_{2}=13,M=20,B=10$	0.6424	0.2736	0.0654

**Table 5:** Estimation results of mutual coupling coefficients.

	Theoretical Value of $c_{b}$	Mean Value of ${\hat{c}}_{b}$	Estimation Biases $\varepsilon_{r}$
$c_{1}$	0.3500 + 0.6062i	0.3484 + 0.6081i	0.0139
$c_{2}$	0.3381 + 0.0906i	0.3391 + 0.0893i	0.0241
$c_{3}$	0.2021 − 0.1167i	0.2000 − 0.1206i	0.0555
$c_{4}$	0.0453 − 0.1690i	0.0529 − 0.1640i	0.0805
